# Do Different Tree-Ring Proxies Contain Different Temperature Signals? A Case Study of Norway Spruce (*Picea abies* (L.) Karst) in the Eastern Carpathians

**DOI:** 10.3390/plants11182428

**Published:** 2022-09-17

**Authors:** Andrei Popa, Ionel Popa, Cătălin-Constantin Roibu, Ovidiu Nicolae Badea

**Affiliations:** 1 National Institute for Research and Development in Forestry ‘Marin Drăcea’, 007190 Bucharest, Romania; 2 Faculty of Silviculture and Forest Engineering, Transilvania University of Brașov, 500036 Brașov, Romania; 3 Center for Mountain Economy (CE–MONT), 725700 Vatra Dornei, Romania; 4 Forest Biometrics Laboratory, Faculty of Forestry, ‘Stefan cel Mare’ University of Suceava, 720229 Suceava, Romania

**Keywords:** climate–growth relationship, climate signal, tree-ring width, basal area increment, blue intensity, daily climatic data

## Abstract

One of the most important proxy archives for past climate variation is tree rings. Tree-ring parameters offer valuable knowledge regarding how trees respond and adapt to environmental changes. Trees encode all environmental changes in different tree-ring parameters. In this study, we analyzed how air temperature is encoded in different Norway spruce tree-ring proxies along an altitude gradient in an intramountain valley of the Carpathians. The study area, in the Gheorgheni region, Romania (Eastern Carpathians), has a mountain climate with a frequent temperature inversion in winter. The climate–growth relationship was analyzed for two contrasting altitudes: low elevation, i.e., below 1000 m a.s.l., and high elevation, i.e., above 1500 m a.s.l. Two local weather stations, one in the valley and the other on the upper part of the mountains, provide daily temperatures (Joseni—750 m a.s.l. and Bucin—1282 m a.s.l.). The bootstrap Pearson correlation between cumulative daily temperature data and three tree-ring proxies (tree-ring width—TRW, basal area increment—BAI, and blue intensity—BI) was computed for each series. The results show that elevation modulates the climate response pattern in the case of BI, and remains relatively similar for TRW and BAI. The winter temperature’s positive influence on spruce growth was observed in both TRW and BAI chronologies. Additionally, the BAI chronology highlights a positive relationship with summer temperature. The highest correlation coefficient (r = 0.551, *p* < 0.05, *n* = 41) was recorded between BI residual chronology from high elevation series and summer/autumn temperature from the upper-part weather station for a cumulative period of 59 days (the second half of August to the beginning of October). Our results show that, for this intramountain valley of the Eastern Carpathians, different tree-ring proxies capture different climate signals.

## 1. Introduction

Tree growth is driven by multiple factors, and climatic conditions represent one of the main drivers of wood accumulation [1,2]. Changes in climatic conditions (e.g., temperature rise, precipitation decrease) and higher frequencies of extreme events (e.g., heat waves, long periods of drought) induce stress on trees and forest stands, with negative ecological and economic effects [3,4]. All these environmental changes are encoded in different tree-ring parameters.

In the context of climate change, increasing efforts have been made to understand and assess the effect of environmental change on forest ecosystems [5]. Knowledge and mitigation of climate-change impacts represent a central goal for forest management systems [6,7,8,9,10].

Dendrochronological studies analyzing how trees are influenced by climate and how tree species adapt to new climate conditions also provide a record of the past climate [1,2,11]. Tree rings can be used as an important proxy to highlight annual climate variations [12]. The main tree-ring proxy used in dendrochronology is tree-ring width (TRW). However, in some cases, TRW does not provide a strong and robust climate signal compared to other tree-ring parameters such as maximum latewood density (MXD) or stable isotopes [13,14,15,16,17,18]. Usually, to ascertain MXD, expensive equipment is required, and there are logistical limitations. A relatively new parameter (image-based blue reflectance—blue intensity, BI) has been developed to respond to these limitations [19,20,21]. BI is a proxy that represents measured reflected light in specific wavelengths of the color spectrum. Studies have shown a strong correlation (over r = 0.95, *p* < 0.05) between BI and MXD [19,20,21,22,23,24]. BI has a stronger climate signal in temperature-limited ecosystems compared to TRW and is less sensitive to disturbances [24,25]. Based on these findings, there is potential for BI to be used as a substitute for MXD. However, the basal area increment (BAI) represents a two-dimensional measurement, specifically on the surface of the tree ring. The basal area increment is more related to the biomass increment of the tree and stand productivity [26,27]. Moreover, BAI is a suitable proxy that can preserve low and medium-frequency growth variability [28,29].

Norway spruce (*Picea abies* (L.) Karst) is one of the dominant coniferous species at both the Romanian and European levels [30]. On a large scale, Norway spruce is managed in monocultures due to its high productivity and ability to grow at high rates inside and outside of natural areas, with significant economic advantages [31]. Monoculture management, which focuses mainly on wood production, is prone to more issues compared to mixed-forest management, which focuses on both productivity and biodiversity, with higher stand resistance and resilience [8,32,33]. Generally, Norway spruce growth is driven by summer temperature in mountainous areas and by precipitation at lower elevations [34,35,36,37,38,39]. Spruce has demonstrated high sensitivity to extreme events such as drought or heatwaves [40,41,42].

In mountainous areas, due to alternating slopes and valleys, it is possible to have a regional climate modulated by a local topography [43]. The particular local climate specific to the intramountain valleys of the Carpathians can induce different climate responses of tree species (in our case, Norway spruce) compared with general patterns observed in mountainous regions.

In this study, we aimed to determine the climate signals captured in three tree proxies (TRW, BAI, and BI) of Norway spruce in an intramountain valley in the Eastern Carpathians along an altitudinal gradient. The specific research questions were:

- How does air temperature modulate Norway spruce growth in an intramountain valley of the Carpathians?
- Is the correlation between temperature and the investigated tree-ring parameters stable through time?

## 2. Results and Discussion

### 2.1. Description of Chronologies

The individual age of sampled trees varies from 68 years to 135 years with a mean chronologies age of 98 years, with insignificant differences between low- and high-elevation chronologies (Table 1). The average growth rate is 1.96 mm·year^−1^, and ranges from 1.77 mm·year^−1^ (high elevation) to 2.16 mm·year^−1^ (low elevation). A reduction in tree growth with increasing elevation is expected, being the consequence of temperature decrease and shortening of the growing season [34,44].

Common variance, expressed by the mean series intercorrelation (Rbar) with values around 0.3 for all tree-ring proxies except for BI at the low elevation, reflects a medium climatic control of Norway spruce growth in the study area. The mean sensitivity of the analyzed chronologies showed lower interannual variability for BI chronologies compared to TRW or BAI chronologies. Low mean-sensitivity values are typical for Norway spruce growing in optimal climatic conditions [35]. Lower values for mean sensitivity of BI chronologies have also been recorded for Norway spruce in other regions [45,46] or for other species from the *Picea*, *Abies* genus [45,47,48]. The temporal memory, expressed by serial autocorrelation of raw data, is highest in the case of TRW and lowest for BI.

**Figure 1:**
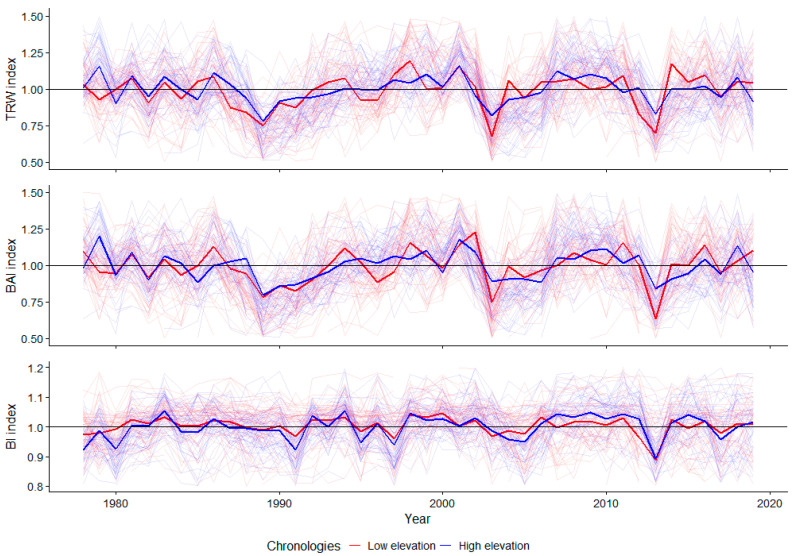
Norway spruce TRW, BAI, and BI residual index series chronologies for 1978–2019 (the shadowed line represents the individual series and the thicker line represents the master chronologies).

The TRW, BAI, and BI index chronologies were truncated for the period 1978–2019 to overlap with the climatic data (Figure 1). The expressed population signal (EPS) for the analyzed period exceeds the threshold of 0.85 [49] for all chronologies.

### 2.2. Climate–Growth Relationships for Three Tree-Ring Parameters

**Figure 2:**
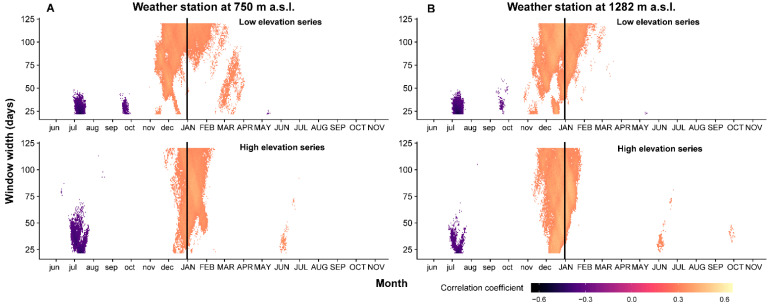
Correlation between TRW residual index chronologies and cumulative daily temperature from valley weather station (Joseni) (**A**) and from up-hill weather station (Bucin) (**B**) (vertical black line represents the limit between previous (with lowercase) and current (with uppercase) year).

The TRW residual index chronologies correlate positively with winter temperatures (cumulative windows width starting from 21 to 120 days) (Figure 2). The correlation coefficient between high-elevation TRW index chronology and mean temperature from the up-hill weather station (Bucin) has the highest value (r = 0.494, *p* < 0.05, *n* = 41) with the 3 December–18 January period. The low-elevation TRW index chronology has the highest correlation (r = 0.485, *p* < 0.05, *n* = 41) with the 1 November–12 February mean temperature from the up-hill weather station. Regarding the correlation between the TRW residuals index and the winter air temperature from the valley weather station, the maximum correlations were lower (r= 0.442, *p* < 0.05, *n* = 41—high-elevation chronology and r = 0.435, *p* < 0.05, *n* = 41—low-elevation chronology).

Moreover, the TRW residual index chronology from low-elevation sites shows a positive and significant correlation with spring temperature (recorded in the valley) from March to April (r = 0.374, *p* < 0.05, *n* = 41). For both chronologies, low and high elevation, a negative correlation between TRW residual index and mean temperatures is present in the previous vegetation season, in July. The maximum negative correlation between TRW residual index chronologies and previous summer temperature varies from r = −0.502 (*p* < 0.05, *n* = 41) for low-elevation series (temperature from the up-hill weather station) to r = −0.462 (*p* < 0.05, *n* = 41) for high-elevation series (temperature from the up-hill weather station). A significant negative correlation between TRW index chronology and temperature in the previous autumn is present only at low-elevation sites, regardless of the weather station.

A positive correlation between the TRW index and December temperatures has also been reported for other forests in the Eastern Carpathians [50]. In mountainous regions, and mainly at high elevation, the growth of Norway spruce is usually positively correlated with summer temperatures [35,37,38,50,51,52]. The positive correlation between TRW index chronologies and winter temperatures at an elevation above 1500 m a.s.l. is not a common dendroclimatic pattern for Norway spruce. The possible explanation for this climate–growth relationship could be related to temperature inversion with a high frequency during winter.

Generally, the air temperature is characterized by a decreasing gradient proportional to the elevation [53]. The average gradient for decreasing air temperature with the increase in elevation, in mountainous areas, is 0.6 °C/100 m [54]. Temperature inversions are phenomena caused by the abnormal variation of the radiative heat balance induced by terrain fragmentation, depth of valleys, and local topographical peculiarities [55,56,57]. Temperature inversions are a common occurrence in intramountain valleys, especially in the Eastern Carpathians [56,58]. Thermal inversion leads to cold-air stagnation at the bottom of the valleys, which favors fog formation with a significant impact on both human health and vegetation growth [59]. At the same time, the upper part of the slopes benefits from higher global radiation and warmer air mass with positive effects on vegetation and soil conditions.

Due to the temperature inversion phenomenon or mild winters, a higher air temperature in the cold season can lead to faster snow melt and higher soil temperature. These factors have a positive influence on cambium reactivation and apical growth in the spring, decreasing the risk of tissue freezing and even creating a low probability of xylem embolism in the spring [60,61,62]. These hypotheses could offer a possible explanation for the positive and significant correlation between temperatures in the winter and TRW index chronologies.

A negative correlation between TRW index series and temperature from the previous vegetation season has been observed in other Eastern Carpathian sites [50], Tatra Mountains [38,63], in the timberline in East-Central Europe [37,64], and in the Alps [65]. This correlation highlights a sensitivity of Norway spruce growth to the previous year’s temperature. Increasing summer temperatures may induce a reduction in the growth of spruce in the next year. This temporal memory of growth can be linked with structural carbohydrate dynamics and extending the growing season in previous years [51], and is supported by high values of first order autocorrelation (Table 1). The correlation of growth with the previous year temperature highlighted the importance of the carryover effects of climate variability, such as photosynthetic gain and storage of assimilates [1,66]. Moreover, a warm summer promotes the flowering of spruce, which is associated with a decrease in growth in the next year [67]. Hansen and Beck [68] highlight that the carbohydrates accumulated in previous autumn are depleted in spring. Also, the dynamics of spruce reserves involve an accumulation of lipids in summer which are metabolized during the autumn [69].

**Figure 3:**
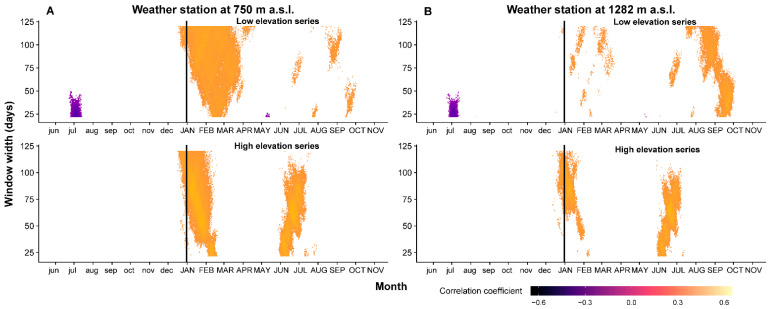
Correlation between BAI residual index chronologies and cumulative daily temperature from valley weather station (Joseni) (**A**) and from up-hill weather station (Bucin) (**B**) (vertical black line represents the limit between previous (with lowercase) and current (with uppercase) year).

The BAI residual index chronology correlation pattern differs depending on elevation (Figure 3). The correlation between high-elevation BAI index chronology and winter temperatures recorded at the up-hill weather station is significant and has a higher value for the period of 26 November to 18 February. Interestingly, the positive correlation between winter–spring temperatures and the BAI index chronology from a low elevation is no longer significant when the temperatures are considered from the up-hill weather station. Regarding the high-elevation BAI index chronology, the correlation patterns with respect to the mean temperatures from both weather stations are similar. In the case of the winter period, the correlation with temperatures from the valley weather station is significant for longer cumulative window lengths.

Compared to the dendroclimatic pattern of TRW, a positive relationship with summer temperature was observed for BAI. The highest correlation was recorded between the BAI chronology from the high elevation and cumulative temperatures from the valley weather station from 17 May to 22 July (r = 0.468, *p* < 0.05, *n* = 41). A positive correlation with summer temperature on the current year’s wood accumulation is logical for high-altitude chronology, since temperature is a limiting factor for these habitats [35]. A negative correlation (r = −0.507, *p* < 0.05, *n* = 41) between BAI residual chronology from lower sites and previous summer temperatures is significant for cumulative periods of 29 days (second half of June to the middle of July) for both weather stations. The authors of [51] point out that higher temperatures during the summer can induce a high rate of respiration with negative effects on carbohydrate reserves used in the first phase of growth of the next year. The photosynthetic gain during the previous summer has a strong effect on current year ring width [67,70].

**Figure 4:**
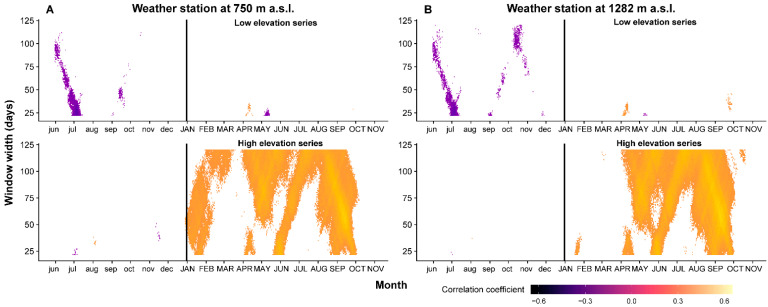
Correlation between BI residual index chronologies and cumulative daily temperature from valley weather station (Joseni) (**A**) and from up-hill weather station (Bucin) (**B**) (vertical black line represents the limit between previous (with lowercase) and current (with uppercase) year).

A clear pattern of positive and significant correlation coefficient between temperature and BI chronologies was found only for the high-elevation chronology (Figure 4). The highest correlation between the BI residual chronology from high-elevation series and summer/autumn from the up-hill station is 0.551, *p* < 0.05, *n* = 41 for cumulative windows of 59 days (second half of August to the beginning of October). An unusual correlation was found between the BI index and winter temperature from the valley weather station. This may be a false-positive correlation because it is less likely that winter temperature has a strong influence on the wood density of latewood. The correlation between low-elevation BI chronology and mean temperature from the previous summer is negative and significant (r = −0.481, *p* < 0.05, *n* = 41) for a cumulative window of 28 days; that is, 19 June–17 July. No significant correlation between BI index and previous year temperature was noted in the case of the high-elevation chronology.

In contrast, at low elevation, the BI chronology shows almost no significant correlation with current year temperatures. This suggests that at elevations below 1000 m, in this intramountain valley, the late-summer temperature is not a limiting factor in the thickening and lignification of cell walls of Norway spruce. This can be linked to a longer growing season at lower-altitude sites and a higher stand productivity [35]. The highest correlation between tree-ring parameters and temperature has been reported for BI chronologies from the high elevation. These BI correlation patterns show that thickening of the secondary cellular wall and the lignification process at high altitude are driven by the late-summer temperature [71]. The negative relationship with the previous year’s temperature can be explained by the trade-offs in the carbohydrate allocation for seed production, increment and formation of buds, with significant effects on nutrients reserves available for next year’s growing start [72,73]. It has already been reported in the literature that BI chronologies, as surrogates for maximum latewood density, express a stronger relationship with climate compared to TRW at sites where the temperature is the most limiting factor [16,20,74,75,76].

Tree-ring width or basal area increment are tree-ring proxies containing aggregated information about the climate conditions throughout the whole growth season, and about disturbances. Meanwhile, the blue intensity contains information about the second part of the growing season. The combination of these three tree-ring proxies can offer an integrated perspective on the climate–growth relationship.

### 2.3. Time Stability of Climate–Growth Relationship

**Figure 5:**
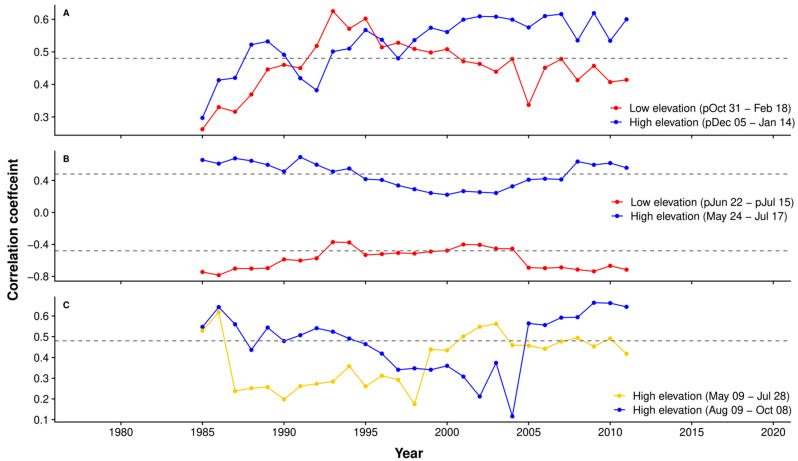
The 15-year running correlations between air temperature and tree-ring proxies ((**A**)—tree-ring width; (**B**)—basal area increment; (**C**)—blue intensity). The legend contains the site location and period (with the dashed line representing a significant correlation coefficient for *p* < 0.05, *n* = 15).

The time stability correlation between TRW, BAI, and BI residual chronologies and temperature from the up-hill weather station was assessed using the cumulative window with the highest correlation coefficient. It was preferred to assess the time stability for windows with the highest correlations despite different periods between low and high elevation chronologies. The periods with the highest correlations may be the most relevant for assessing the climate sensitivity of Norway spruce. In the case of TRW, for the low-elevation series, the highest correlation was for the interval between 31 October and 18 February and, in the case of the high-elevation series, for the period between 5 December and 14 January. The time variation pattern of the correlation has evident temporal shifts, mainly in the second part of the analyzed period (Figure 5A). For the high-elevation chronology, the correlation has an increasing general trend with a small decrease around 1992 and stabilization after 2000. Contrary to the low-elevation chronology, the correlation intensity increased until the 1989–2003 period. After that, a slight decrease was recorded, with robust stability in time in the last years. The correlation between the low-elevation TRW chronology and temperature from the up-hill weather station is lower than the correlation recorded for high-elevation sites for most of the periods analyzed.

The stability of correlation between the BAI chronology from upper sites and growing season temperatures (24 May to 17 July) is stable in time with a slight decrease around the 2000s (Figure 5B). The correlation coefficient for the entire period is r = 0.441, with a minimum correlation of r = 0.298 for the period 1995–2009. Similarly, for lower sites, the BAI chronology moving correlation with previous summer temperatures (22 June to 15 July) records a reduction of the intensity in the middle of the analyzed periods, which is statistically non-significant. The decrease in correlation highlights a temporal instability in the climate sensitivity of BAI for both elevations, which has also been reported in other Norway spruce sites around Europe [34,44].

The time stability for BI correlation with air temperature was checked only for the high-elevation chronology (Figure 5C). Two periods were analyzed, one in the first part of the growing season (9 May to 28 July) and one in the late part (9 August to 8 October). The correlation between the temperature at the beginning of the growing season and BI index chronology has lower values in the first part of the analyzed period. The correlation is stable in time and significant only after the 1997–2010 period. The temperature from the last part of the growing season leads to a constant decrease in time of the correlation with the BI residual chronology, followed by a significant increase after 2004.

The running correlation for a 15-year period highlights the non-stationary correlation between TRW, BAI, and BI index chronologies, and air temperature. The continuous climate change leads to different responses of trees to temperature.

## 3. Materials and Methods

### 3.1. Study Area

**Figure 6:**
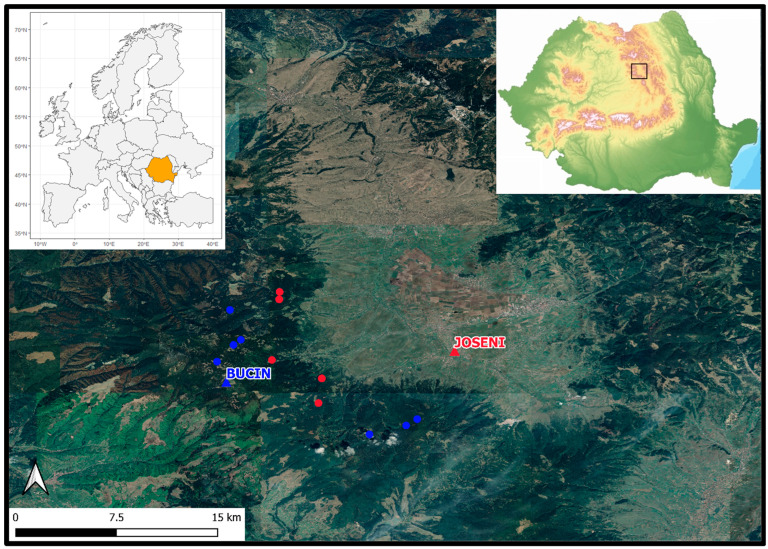
The study localization—with blue the high elevation sites, up-hill weather station, and with red the low elevation sites, valley weather station (image source: https://geoportal.ancpi.ro (accessed on 1 August 2022); https://earth.google.com (accessed on 1 August 2022)).

The study was carried out in the Gheorgheni region, a large intramountain valley in the center of the Eastern Carpathians (Romania). The altitude in the study region ranges from 700 m a.s.l. to 1770 m a.s.l. The study area is located between 46°37′ N, 25°25′ E and 46°50′ N, 25°36′ E (Figure 6). The general geology of the study area is represented by a volcanogenic–sedimentary complex.

All the forests in the study area are managed forests, even-aged, and the Norway spruce is the main species. The forests belong to private and community owners and are managed by Gheorgheni Forest District manly in a high forest silvicultural system (clear-cutting system and shelterwood system).

**Figure 7:**
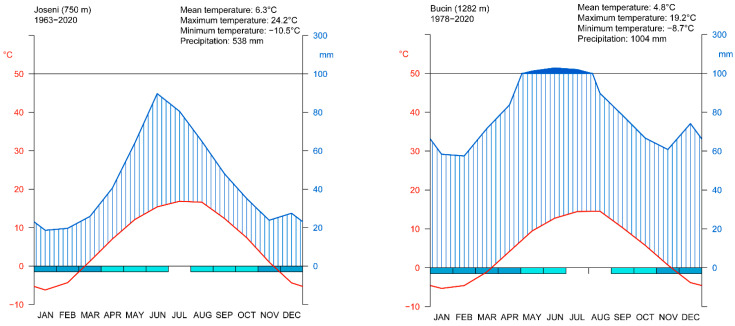
Climate diagrams according with Walter and Leith [77] for the local weather stations in the study area (valley station on the right and up-hill station on the left). The red line represents the variations of the mean monthly temperatures, the blue line represents the variations of the monthly precipitation, the blue hatched area represents the humid period, and the blue filled area represents the wet period. The bar in the lower part suggests the indication of months where frost is likely.

In the study area mean annual temperature varies from 6.0 °C at the lower part of the valley to 4.8 °C at higher elevations (Figure 7). The average amount of annual precipitation is 542 mm at 750 m a.s.l. and 1004 mm in the upper part of the valley. The coldest month is January (the mean temperature at the lower part of the valley is −6.4 °C and −5.7 °C at higher elevations) and the warmest is July (the mean temperature at the lower part of the valley is 16.6 °C and 14.1 °C at up-hill weather station). The month with the most precipitation is June with 130 mm in the upper part of the valley and 90 mm at the lower part. The study area is characterized by frequent thermal inversion [59]. January is the month with the highest average of days with temperature inversion (13.8 days) followed by December (12.9 days) and November (11.9 days). During the spring/summer months, on average, less than 1–2 days with temperature inversion were recorded.

The climate diagrams according to Walter and Leith were created in R using the climatol package [78].

### 3.2. Sample Collection and Data Processing

To investigate the effect of altitude on the Norway spruce, climate response increment cores from 12 locations were collected: five locations at low elevation (altitude varied from 880 m a.s.l. to 1020 m a.s.l.) and seven locations at high elevation (altitude varied from 1510 m a.s.l. to 1630 m a.s.l.). In each plot, 15 to 20 dominant and healthy trees were selected to extract increment cores. By applying a sampling strategy, we selected mature trees, and the limitation of the analysis period to available temperature data ensured the exclusion of the juvenile growth part from the climate–growth relationship analysis. One core per tree was extracted using a 5-mm-diameter Pressler borer at breast height (i.e., 1.3 m). For each sampled tree, 2 perpendicular breast-height diameters (DBH) were measured with a forest caliper. The mean of these two diameters was used to compute the BAI. All three tree-ring proxies (TRW, BAI, and BI) were measured/calculated for the same cores. A subset of 50 cores for each altitude level (high and low elevations), were selected for measurements and analysis. In order to obtain reliable BI measurements, we selected only cores with no discoloration due to fungi, no gaps due to broken cores, and with parallel rings, as well as from cores containing pith or those that allowed easy determination of any missing rings.

Increment cores were dried, mounted on wooden supports, and sanded with successive sanding grits (from 80 grit to 400 grit) to ensure the flatness of the sanded surface and ring boundaries’ visibility. Sandpaper of 400 grains/mm^2^, according to [79], can assure the quality of BI measurements. After sanding, cores were scanned at 1200 DPI using an Epson Expression 12,000 XL scanner to measure TRW and BI. The scanning resolution (1200 DPI) used in this study represents the scanner’s hardware optical resolution, and we did not use an interpolation algorithm that could affect the BI measurements [79].

Norway spruce is a coniferous species with no visible differences between heartwood and sapwood. Based on this characteristic of spruce wood, it is possible to measure latewood blue reflectance without any chemical treatments [22,80,81,82]. To measure latewood blue reflectance, the standard protocol was followed [21,23]. Window parameter settings were adjusted according to [21]. The measured values of blue reflectance were transformed into blue intensity (BI) using Equation (1):

(1) $$x_{i\left(adj\right)}=2.56-\;\frac{x_{i}}{100}$$

where *x_i_* represents the raw BI value for the year *i*; the constant 2.56 is used to ensure that all the values of BI are positive (all the possible values for *x_i_* are from 0 to 255). The inversion step was computed in CooRecorder 9.6 [83].

The tree-ring measurements (TRW and BI) were computed using CooRecorder software on scanned images [83]. No missing or false rings were observed.

The BAI was reconstructed for each ring individually and adjusted according to the mean DBH of each tree. The DBH of each tree (*i*) and for each year (*t*) was reconstructed by subtracting the doubled radial growth in the year *t* from the DBH in the year *t*. The calculation starts with the DBH measured in the year of coring (Equation (2)) [84].

(2) $$DBH_{i\left(t-1\right)}=DBH_{i\left(t\right)}-2\times TRW_{i\left(t\right)}$$

Based on the annual reconstructed DBH, the BAI for each tree was calculated using the following equation:

(3) $$BAI_{t}=\frac{\pi }{4}\times \left(DBH_{t}^{2}-DBH_{t-1}^{2}\right)$$

The BAI was calculated using an iterative function in R [85].

The measured individual series were cross-dated visually using TSAP-Win software [86]. COFECHA software was used for statistically cross-dating and measurement checking using correlation analysis of detrended 50-year intervals with a 25-year overlap [87,88].

A cubic smoothing spline function with a 50% frequency cutoff at 30 years was used to eliminate the age trend and any other disturbance signals in TRW and BAI chronologies [89,90]. According to [21], for the BI value, there is a possible increase in the juvenile period due to the transformation from blue reflectance to blue intensity. Because of this, the detrending of the BI series was made by fitting a Hugershoff curve to raw measurements [89].

For each analyzed tree-ring parameter, 2 chronologies were developed, one for low elevation and the second for high elevation. Indices were computed as the ratio between raw measurements and fitted values. In order to eliminate the autocorrelation that was still present in the standard index series, an autoregressive model was applied. In the analyses, we used the residual index chronologies obtained by bi-weight mean without variance stabilization. The chronologies quality and proprieties were assessed by calculating the classical dendrochronological statistics parameters as mean sensitivity, mean series intercorrelation, first-order autocorrelation, and expressed population signal. The mean sensitivity highlights the change in year-to-year tree growth [1]. The mean series intercorrelation (known as R-bar) and expressed population signal (known as EPS) express the signal strength in the analyzed chronologies and the common variability [49,89]. The carry-over effect of the previous year’s climate condition on the current year’s growth can be assessed by first-order autocorrelation [1,2]. The detrending, chronology development, and statistical parameters were computed using the dplR package on R software [91].

### 3.3. Climatic Dataset

Two local weather stations, Joseni (750 m a.s.l.)—valley weather station—and Bucin (1282 m a.s.l.)—up-hill weather station—provided the climatic data (daily mean temperature) (Figure 6 and Figure 7). Climatic data are available from 1963 at the Joseni weather station and from 1978 at the Bucin weather station. Therefore, the analyzed interval was limited to the common period, 1978–2019.

### 3.4. Climate–Growth Relationship Assessment

The availability of local daily air temperature data in the study area allowed us to analyze the climate–growth relationship on a cumulative daily scale [92]. Using the monthly temperature data, a common practice in dendroclimatology [89], to investigate the relationship between climate and tree growth conduce to a signal lost because of the artificial split of year in months, without any physiological bases [92,93]. The tree photosynthesis and wood increment are continuous processes and require more flexible time windows to investigate the relationship between tree growth and climate. The bootstrapped (*n* = 1000 replications) Pearson correlation between the TRW, BAI, and BI residual index chronologies and cumulative daily mean temperature was calculated [94,95]. Bootstrap samples were selected randomly with replacement from the entire interval (1978–2019) [96]. The significance of the bootstrapped correlation coefficient, at *p* < 0.05, was established according with [97]. The daily temperatures were aggregated in moving time windows of 21 days to 120 days starting from June in the previous year of growth to October of the current growing season. The time stability of correlations was checked for running windows with a length of 15 years, moved by one year [96]. The correlation between residual index chronologies and cumulative daily temperatures and time stability were computed using the dendroTools package in R [98].

## 4. Conclusions

Our results show that each tree-ring proxy contains a different climate signal. In the studied intramountain valley in the Eastern Carpathians, the growth of Norway spruce is influenced by winter temperatures, and signals demonstrating this are present in both TRW and BAI chronologies. The presence of temperature inversions can explain the unusual correlation pattern. The TRW and winter temperature correlation is unstable through time for both elevations, with evident temporal shifts after 1996. The highest correlations for TRW were obtained for cumulative windows of 45 days at high elevation and for cumulative windows of 113 days for low elevation; both cumulative windows are centered at the beginning of January. The BAI at high elevation contains a strong temperature signal from the middle of the growing season. In the case of BI, a clear climatic signal was observed only for the high-elevation chronology for cumulative windows starting in the second half of August to the beginning of October. The preliminary results obtained in this study need to be replicated for other intramountain valleys in the Carpathians to confirm the change in the general dendroclimatic pattern (growth driven by summer temperature) due to specific local climate modulated by thermal inversions.

## Figures and Tables

**Table 1:** Basis statistics for the six chronologies: TRW—tree-ring width; BAI—basal area increment; BI—blue intensity; AGR—average growth rate (mm year^−1^ for TRW, mm^2^ year^−1^ for BAI, and no unit for BI); SD—standard deviation; Rbar—series intercorrelation; Mean sens—mean sensitivity; Auto corr.—serial autocorrelation.

Tree Ring Proxy	Series Length			AGR ± SD	Rbar	Mean Sens	Auto Corr.
	Mean	Min	Max				
**Low Elevation Chronologies**							
TRW	95	79	135	2.16 ± 0.53	0.304	0.176	0.869
BAI				1445.7 ± 614.7	0.299	0.211	0.773
BI				2.27 ± 0.17	0.146	0.063	0.548
**High Elevation Chronologies**							
TRW	101	68	133	1.77 ± 0.54	0.335	0.150	0.857
BAI				1003.6 ± 541.6	0.333	0.163	0.844
BI				2.15 ± 0.20	0.287	0.077	0.459

## Data Availability

The data presented in this study are available on a reasonable request from the corresponding author.
